# A Tuberculin Skin Test Survey and the Annual Risk of *Mycobacterium tuberculosis* Infection in Gambian School Children

**DOI:** 10.1371/journal.pone.0139354

**Published:** 2015-10-14

**Authors:** Ifedayo M. O. Adetifa, Abdul Khalie Muhammad, David Jeffries, Simon Donkor, Martien W. Borgdorff, Tumani Corrah, Umberto D’Alessandro

**Affiliations:** 1 Disease Control and Elimination Theme, Medical Research Council Unit-The Gambia, Atlantic Boulevard, Fajara, PO Box 273, Banjul, The Gambia; 2 Academic Medical Centre, University of Amsterdam, Amsterdam, Netherlands; Statens Serum Institut, DENMARK

## Abstract

**Background:**

A Tuberculin skin test (TST) survey was conducted to assess the prevalence of latent TB Infection (LTBI) and to estimate the annual risk of *M*. *tuberculosis* infection (ARTI) in Gambian school children. The results are expected to contribute to understanding of Tuberculosis epidemiology in The Gambia.

**Methods:**

This was a nationwide, multi-cluster survey in children aged 6–11 years. Districts, 20 of 37, were selected by probability proportional to size and schools by simple random sampling. All TST were performed using the Mantoux method. Height and weight measurements were obtained for all participants. We calculated prevalence of LTBI using cut-off points of 10mm, the mirror and mixture modelling methods.

**Results:**

TST readings were completed 13,386 children with median age of 9 years (interquartile range [IQR] 8–10 years). Mixture analysis yielded a cut-off point of 12 mm, and LTBI prevalence of 6.9% [95%CI 6.47–7.37] and the ARTI was 0.75% [95%CI 0.60–0.91]. LTBI was associated gender and urban residence (p <0.01). Nutritional status was not associated with non-reactive TST or sizes of TST indurations. ARTI did not differ significantly by age, gender, BCG vaccination or residence.

**Conclusions:**

This estimates for LTBI prevalence and ARTI were low but this survey provides updated data. Malnutrition did not affect estimates of LTBI and ARTI. Given the low ARTI in this survey and the overlapping distribution of indurations with mixture modelling, further surveys may require complementary tests such as interferon gamma release assays or novel diagnostic tools.

## Background

Population-based annual risk of TB infection (ARTI) derived from Tuberculin skin test (TST) surveys among children is an epidemiological index related to the intensity of community TB transmission. [1] Young children are the preferred target group for TST-surveys as their ARTI estimates correlate better with the recent TB situation in the target community; and sequential ARTI estimates from repeated surveys may be useful for measuring the trend of TB transmission.[2, 3]

The WHO conducted first surveys of TB infection (LTBI) and disease in The Gambia in 1958 and 1960 respectively.[4, 5] In addition, there have been other national/subnational surveys in 1976[6], 1984[7], and 1991[8]. Gambia has not conducted any other survey since the National Leprosy and Tuberculosis Programme was established in 1984. Because earlier survey data are old and guidelines and recommendations for conduct of TST-surveys have changed, it was important to assess the recent burden of LTBI and estimate ARTI.

Therefore, we set out to determine the prevalence of LTBI, estimate ARTI, and the effect of nutritional status on LTBI prevalence estimates in a representative sample of schoolchildren. We aimed to produce a reliable baseline against which to compare trends in transmission and evaluate disease control efforts in the future.

## Methods

### Study setting and population

#### Setting

The Gambia is the smallest mainland country in sub-Saharan Africa with a population of 1.88 million [9] as well as one of the poorest. [10] It has a low HIV prevalence (1.2% [0.8%–1.7%]). [11]

#### Population

This study conducted in 2011, was a nationwide cross-sectional study of primary school children aged 6–11 years (i.e. students in grades 1–4). BCG vaccination is given at birth or as early as possible within the first month of life and national BCG vaccine coverage has been >90% since 1990. [12] So all age eligible children irrespective of BCG scar status were included.

#### Sampling

A final sample size of 12,096 was obtained from a calculated simple sample of 10,080 based on the following assumption; 2% precision for a baseline LTBI prevalence of 10%, cluster coefficient of variation (k) of 40% with a 95% confidence interval (CI) of the between cluster prevalence of 10% ± 8%, 20% inflation to account for exclusions, dropouts and errors. In addition, if the survey is repeated 5 years later, the sample size will be able to detect a 2.5% decrease in the prevalence of LTBI at the 5% significance level [1]

**Fig 1:**
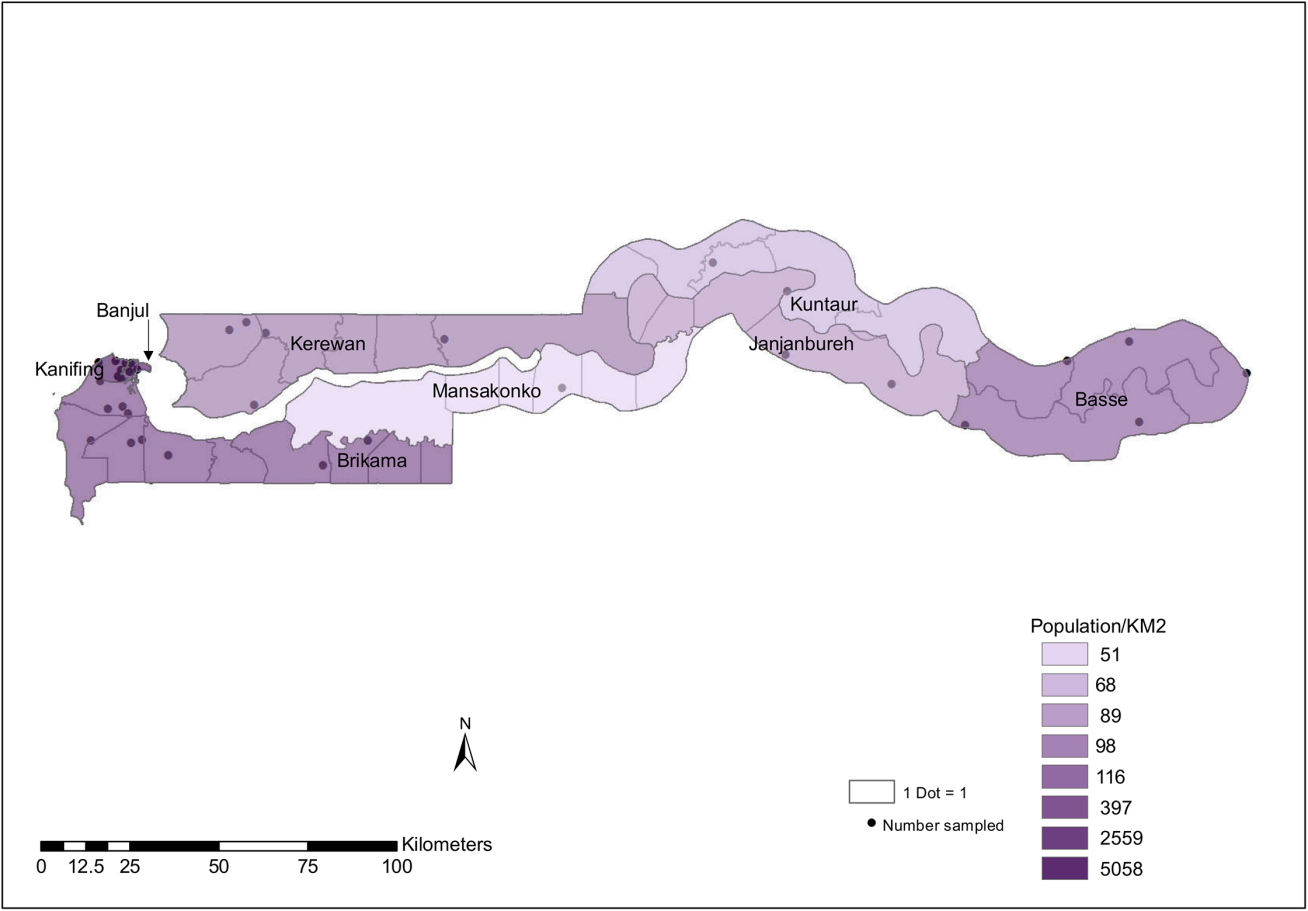
Map of The Gambia showing administrative boundaries, population density and districts surveyed and number of schools surveyed per district.

We followed a 2-stage process for countrywide sampling. First, we randomly selected districts by probability proportional to size (PPS) sampling, which yielded 20 of the country’s 37 districts (Fig 1). Then, we selected lower basic schools by simple random sampling within the selected districts (Fig 1). In the selected schools, all available age-eligible children present on the day of testing were included. The survey team did not perform any further sampling within the schools. Exclusion criteria were, children of non-consenting parents/guardians, those with a significant skin rash (emphasis was on active weepy lesions or those sufficiently generalized to preclude placing the TST), those with history/evidence of recent TST, reported fever or obvious ill health on the day of testing and those receiving TB treatment.

### Field procedures

#### Participant information

Children’s ages and/or dates of birth, gender and residence were copied from the school register or if not available, obtained from the teacher after asking the child. Gambian primary schools serve a range of well-defined catchment communities that students come from.

#### BCG scar examination

The upper third of the left arm of every child was physically examined first then the same location on the right arm for presence/absence of a typical BCG scar. Where there was an atypical scar, the status was recorded as unclear.

#### Anthropometry

Heights were measured in centimetres with SECA Leicester mobile stadiometer and weights with Tanita® digital scale.

#### Tuberculin skin testing (TST)

The survey team received training in testing, reading and scar examination to ensure standardisation prior to the start of the survey. A reference nurse from the regional centre of excellence, Stellenbosch University, South Africa delivered this training. We combined this training with a pilot survey in known TB cases and schoolchildren following which testers and readers for the survey were selected on the basis of their training evaluation reports.

All TST were performed using tuberculin, 2 TU in 0.1ml purified protein derivative (PPD) RT23/Tween 80 (Statens Serum Institut, Copenhagen) with the same batch number for the whole survey. [1, 13] The diameters of a palpable induration were measured after 48–72 hours using a transparent ruler hours. [1, 13]

#### TST cut-offs for diagnosis of LTBI

We looked for a bimodal distribution of tuberculin reactions with a view of using two TST cut offs-10 and 15 mm to estimate the prevalence of infection as done in other surveys.[14] Since we did not observe a bimodal distribution, the presence of a clear mode of tuberculin reactions was sought to estimate prevalence of infection using the mirror method. The mirror method assumes tuberculin reactions are normally distributed around the mode for infection due to *M*. *tuberculosis*. [15] In absence of a clear mode, the mode of reaction sizes of smear positive pulmonary TB cases with definite infection was used to assign a cut-off. The distribution of TST induration sizes in this group is assumed to correlate closely with the distribution in children with LTBI. [1]

Unlike the case in clinical settings where fixed TST cut-offs are utilised for decision-making based on patient risk categories, TST cut-offs for use in surveys such as this are to be derived from the observed distribution of TST induration sizes. [1]

Furthermore, national guidelines do not prescribe a TST cut-off for diagnosing LTBI. [16] Similar to other surveys, [14, 17, 18] we used a fixed cut-off- 10 mm TST and cut-offs derived from the mirror method and mixture model to estimate the prevalence of infection. [19]

### Data management and analysis

Data were double entered, managed with Microsoft Access (Microsoft Corporation, Redmond, WA., USA) and statistical analyses performed using Stata Version 12 (Stata Corp, College Station, TX., USA). The key outcomes were the prevalence of LTBI and ARTI in Gambian schoolchildren.

We examined frequency distributions of TST indurations for terminal digit preference and calculated this as the ratio of the observed Mantoux test reading against a smoothed kernel. The effect of 1000 different bandwidths was investigated (range of bandwidth: 0.5 to 3) to explicitly illustrate digit preference.

Data analysis with and without smoothing to reduce the influence of digit preference on the estimate of prevalence of infection was performed as recommended. [1, 20] We used the Kolmogorov-Smirnov test for equality of distributions to assess the presence of a difference in the distribution of TST indurations (mm) by BCG scar status. We determined which method of deriving a TST cut off provided the best estimate of LTBI prevalence using the Akaike’s Information Criterion (AIC). For each TST threshold, prevalence and ARTIs were calculated and as well as Clopper-Pearson exact confidence intervals of the ARTIs. [21, 22] Connected graphs stratified by locality were used to illustrate the distribution of TST indurations stratified by BCG scar status.

For each TST threshold, univariable and multivariable analyses on the covariates (i.e. age, BCG scar, sex and locality) were performed with logistic regression modelling.

We calculated height-for-age (HAZ), weight-for-age (WAZ) and BMI-for-age (BAZ) in STATA using the WHO AnthroPlus software based on the 2007 growth reference for children and adolescents aged 5–19 years (or 61–228 months). [23] Children with WAZ <-2SD were defined as underweight and as severe underweight at <3SD. Because the pubertal growth spurt may give the impression of excess weight (by WAZ) when children are just tall, WAZ reference data are not available beyond age 10 years.[24] Stunting and severe stunting were defined as HAZ <-2SD and <-3SD respectively. BAZ, >+1SD, >+2SD, <-2SD and <-3SD were the cut-offs for definitions of overweight, obesity, thinness and severe thinness respectively. WHO AnthroPlus automatically flagged extreme z-scores, which were then not used in the nutritional analyses.

We performed significance testing for differences between proportions of the different nutritional indices by location, and by categories of tuberculin reactions (non-reactive [0 mm], reactive [>0 but <10 mm] and positive [≥10 mm]). And performed unadjusted and adjusted logistics regression analyses for associations between nutritional status and the three TST cut-offs.

#### Estimating of the annual risk of M. tuberculosis infection (ARTI)

ARTIs were estimated using the formula below and applying the prevalence estimates for LTBI obtained using the cut-offs and the mixture modelling.

$$\mathrm{ARTI}=1-{\left(1-p\right)}^{1/\left(a+0.5\right)}$$ (1)

**a:** is the mean age of the children in years (0.5 years was added to the mean age calculated from the survey data since age was recorded in full years at the last birthday)

**p:** is the prevalence of infection at the time of survey

### Ethics statement

The Joint Gambia Government/Medical Research Council Ethics Committee approved the survey protocol. Written consent for study procedures was obtained from the parent or guardian of every child.

We considered children with a positive TST (≥ 10 mm) as potentially infected. Those assessed to have clinical symptoms suggestive of active TB in addition to a positive TST (≥ 10 mm) and/or history of recent TB contact was referred for further investigation. The Gambian TB programme has a policy of TB preventive treatment for only under 5-year old children.

## Results

### Study Population

**Fig 2:**
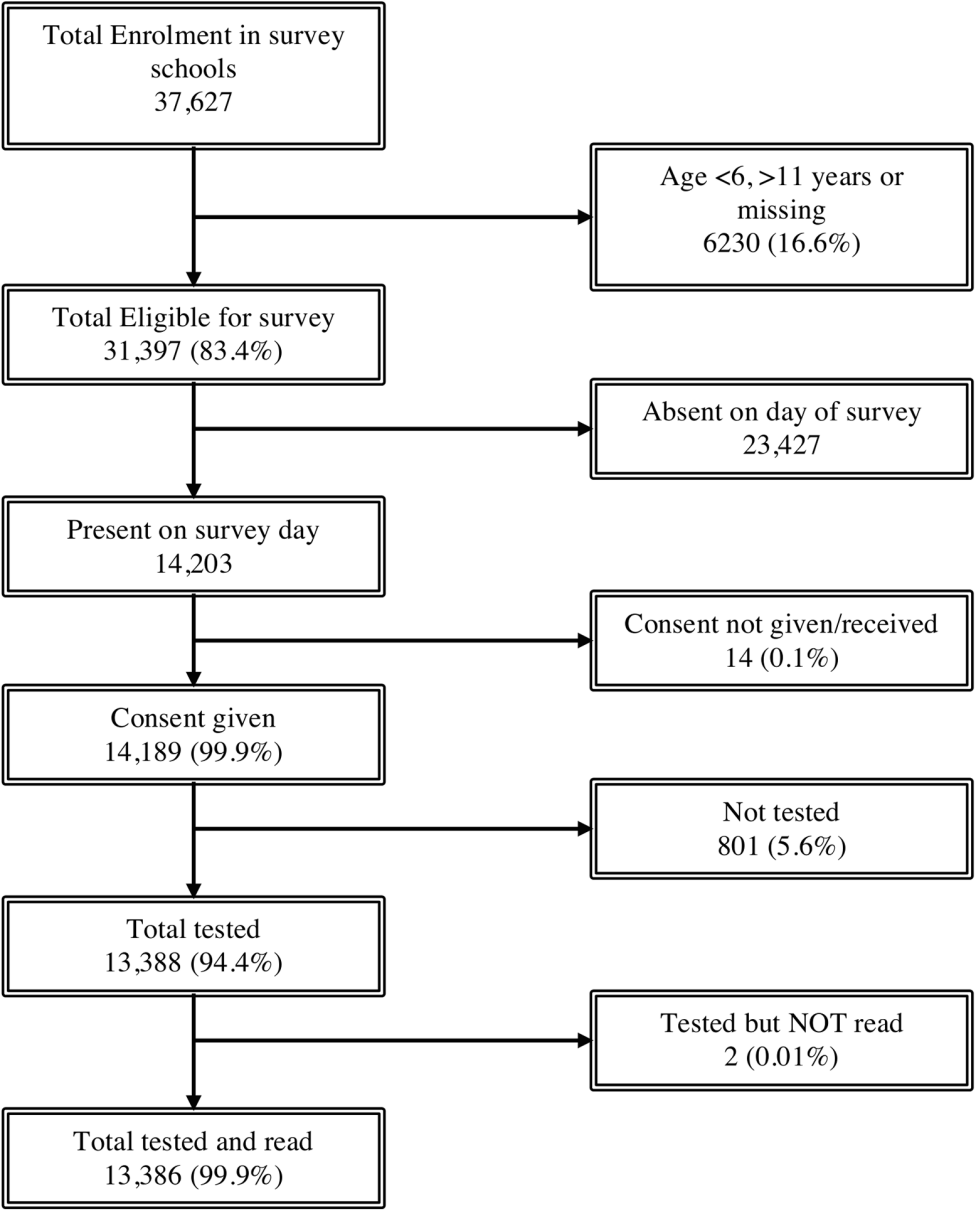
Study Flow Diagram.

Participants were predominantly female (7052 of 13386, 52.7%) and the median age was 9 years (interquartile range [IQR] 8–10 years). The study flow chart is shown in **Fig 2**.

### TST reactions

**Fig 3:**
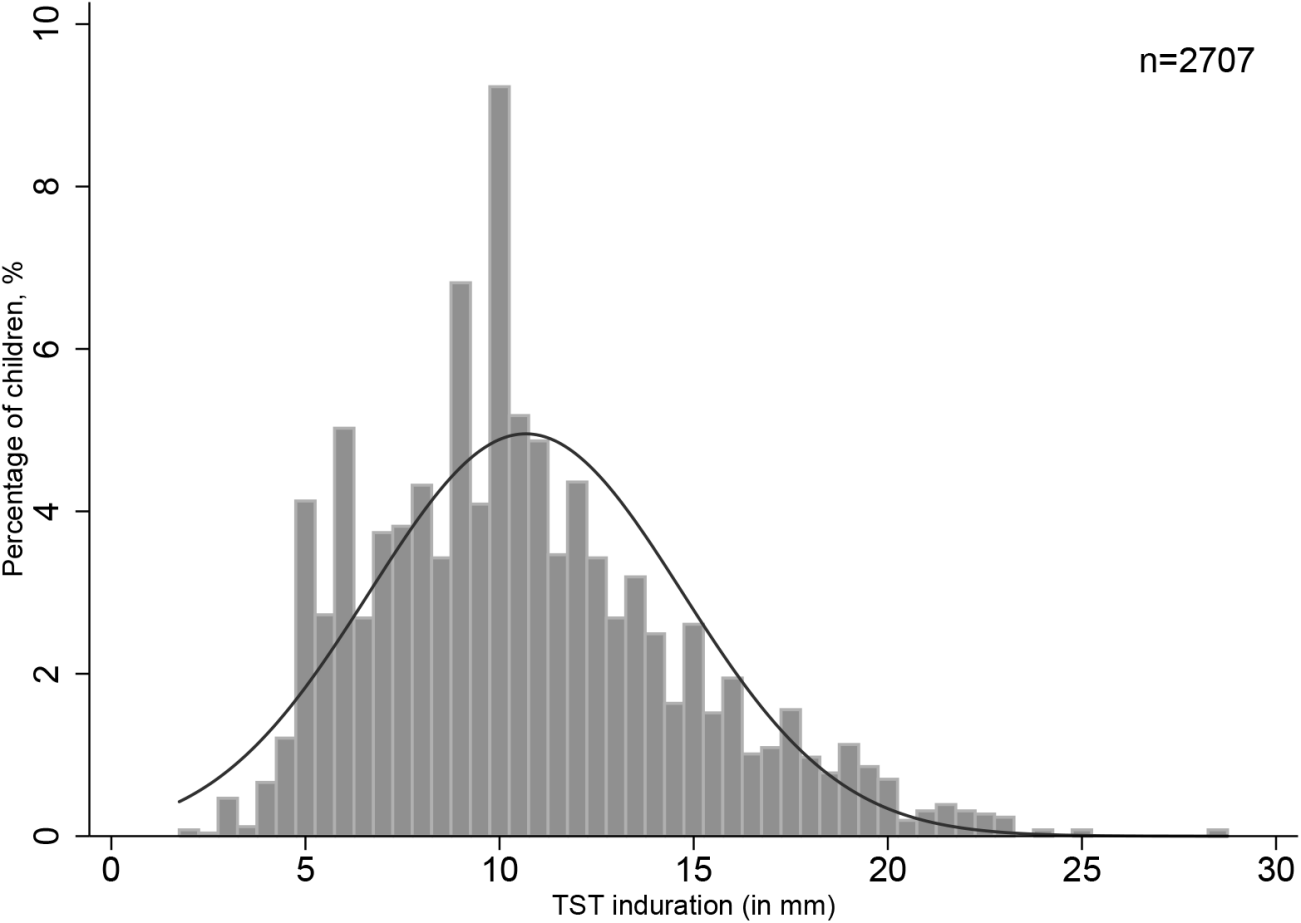
Frequency distribution of tuberculin induration sizes (mm) among Gambian School Children aged 6–11 years.

The frequency distribution of reactions sizes for all survey children with TST indurations >0 mm is shown in **Fig 3**. Majority of survey participants (10,679 of 13, 386 children, 79.8%) had a TST induration readings of 0 mm and two had readings at or above 29 mm. Digit preference was 1.75 and was only seen for 10mm sized reactions.

**Fig 4:**
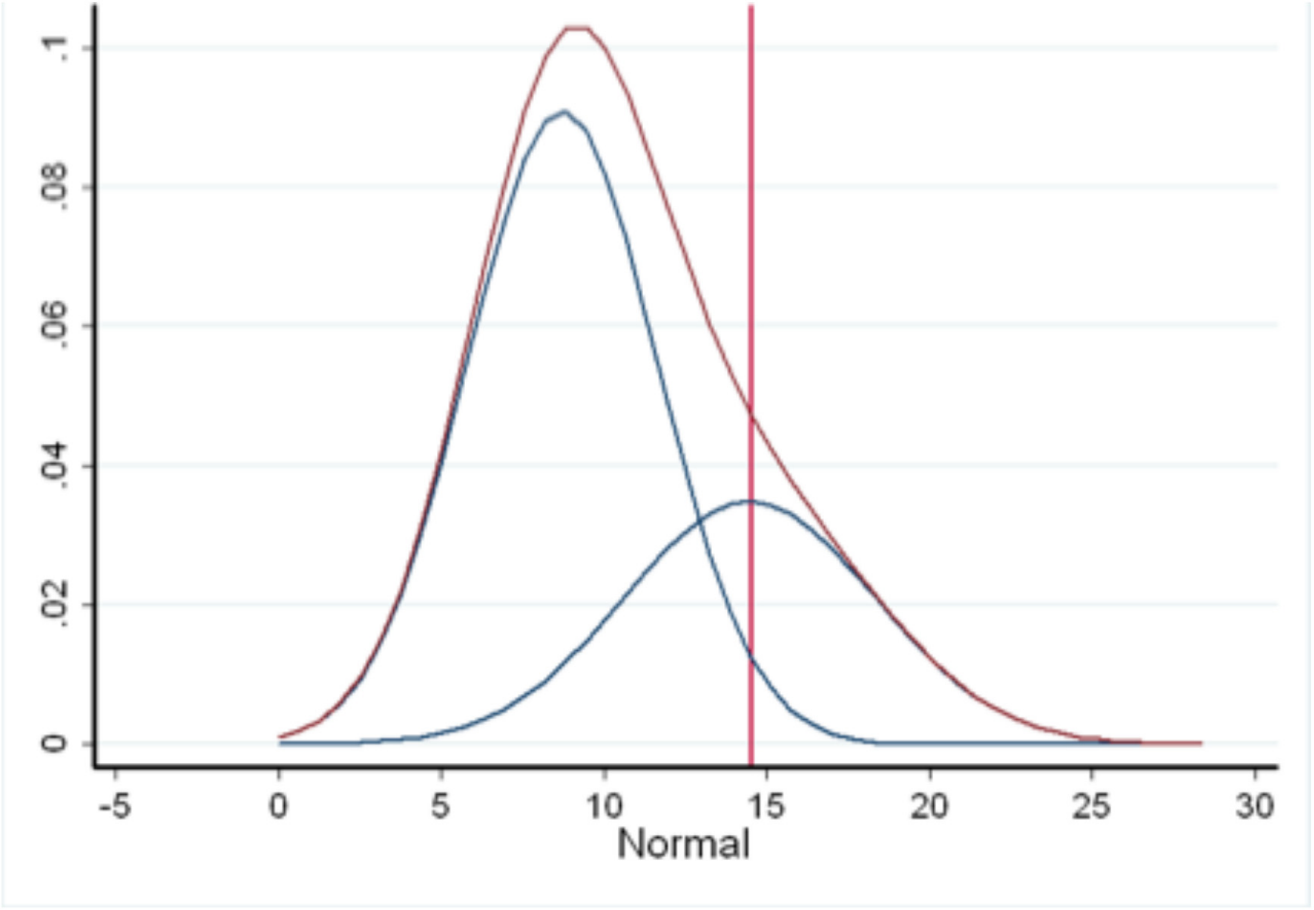
Frequency distribution of smoothed tuberculin induration sizes (mm).

**Fig 5:**
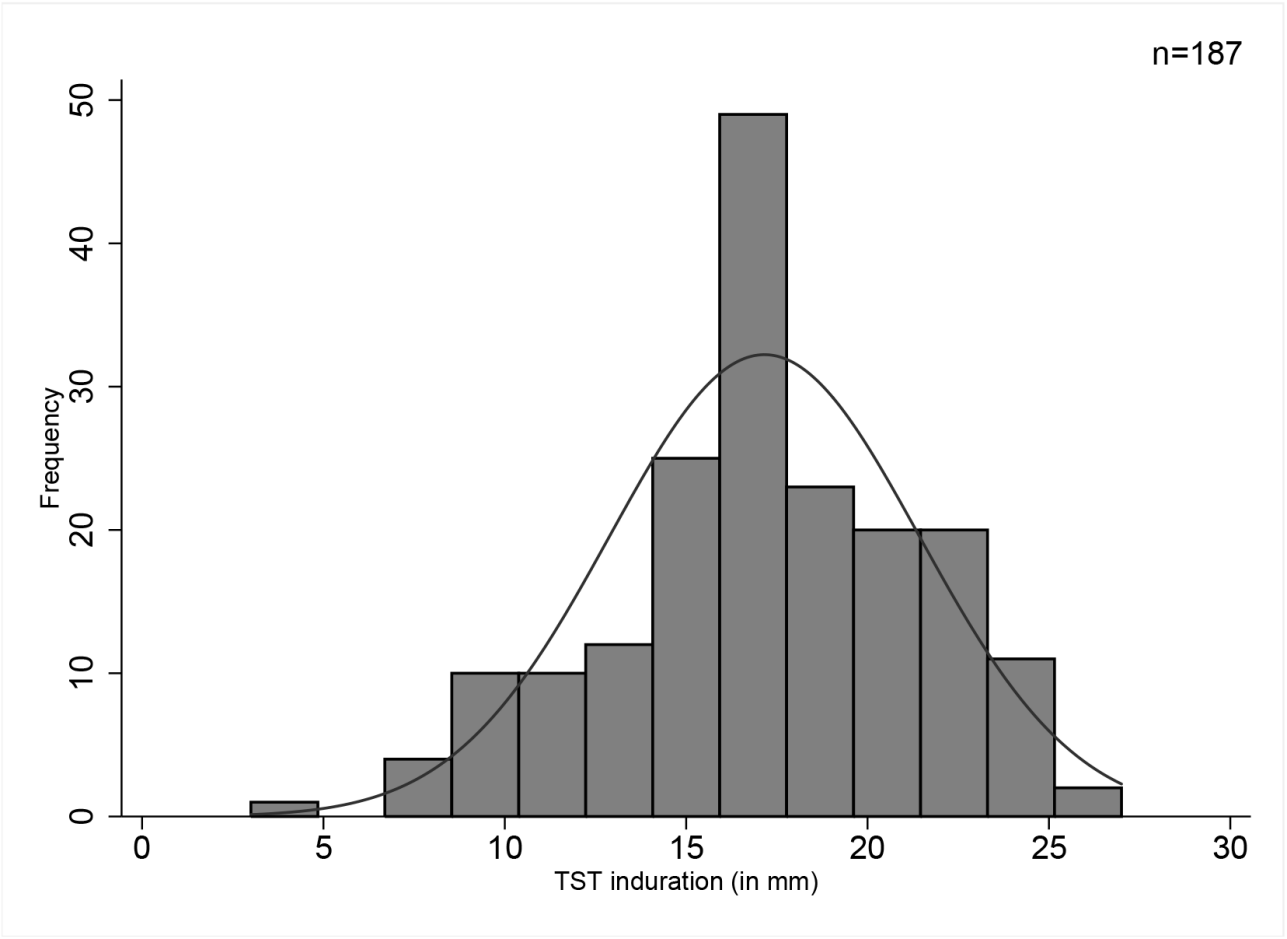
Frequency distribution of tuberculin induration sizes (mm) in a sample population with confirmed Tuberculosis.

The smoothed TST distribution in **Fig 4** yielded two larger modes (9.5 and 10 mm) i.e. the higher peaks instead of a single conclusive mode while **Fig 5** displays the TST distribution in a sample of confirmed TB patients from which we derived an alternative mode. Assuming normality (normality test, p = 0.02), the mean of this distribution was 17.2 mm with a median of 17.5 mm and a mode of 17.5 mm (rounded to 17 mm).

**Fig 6:**
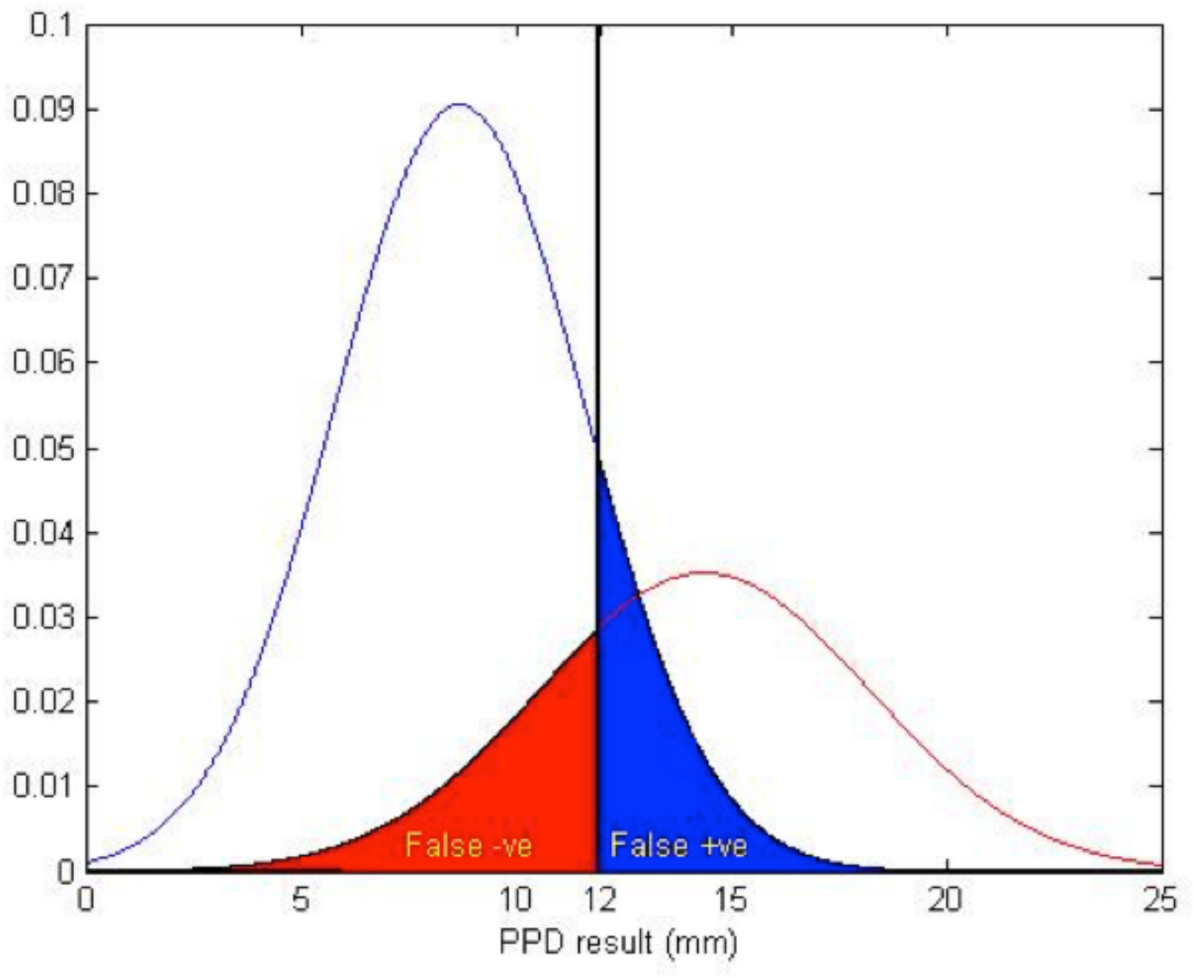
Derivation of TST cut-off from mixture modelling of the frequencies of tuberculin indurations (mm).

Following application of mixture modelling to the smoothed distribution of tuberculin reactions, we obtained the distributions seen in **Fig 6**. With the relatively large overlap between the two composite distributions corresponding to false positive and false negative tuberculin results, a 12 mm TST cut-off equated the probability of false positives/negatives as illustrated in **Fig 6**.

### BCG scar

**Fig 7:**
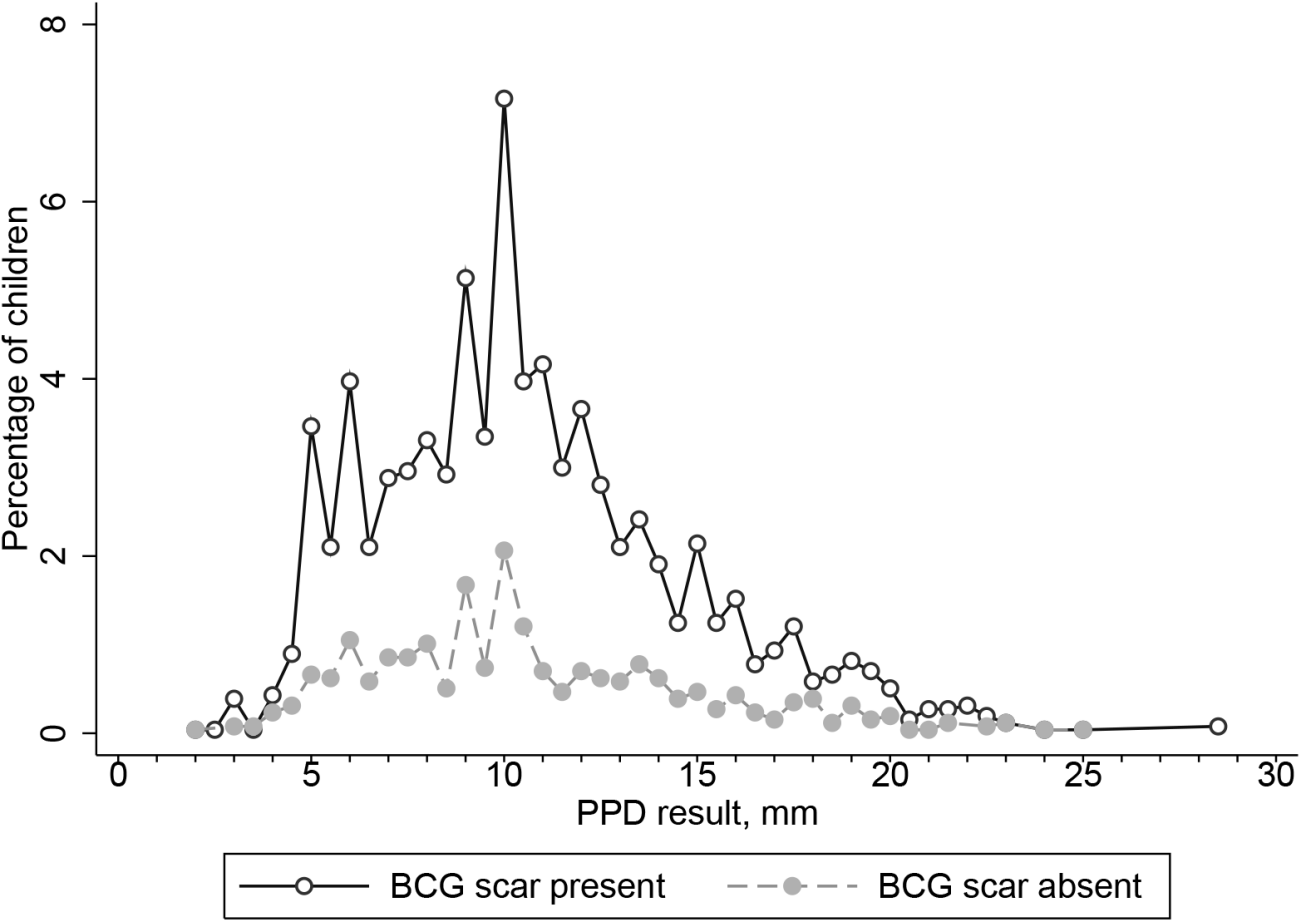
Frequency distribution of tuberculin induration sizes (mm) among Gambian School Children aged 6–11 years by BCG scar status. 10,679 of 13, 386 children, 79.8% had 0 mm induration and are not shown.

**Fig 8:**
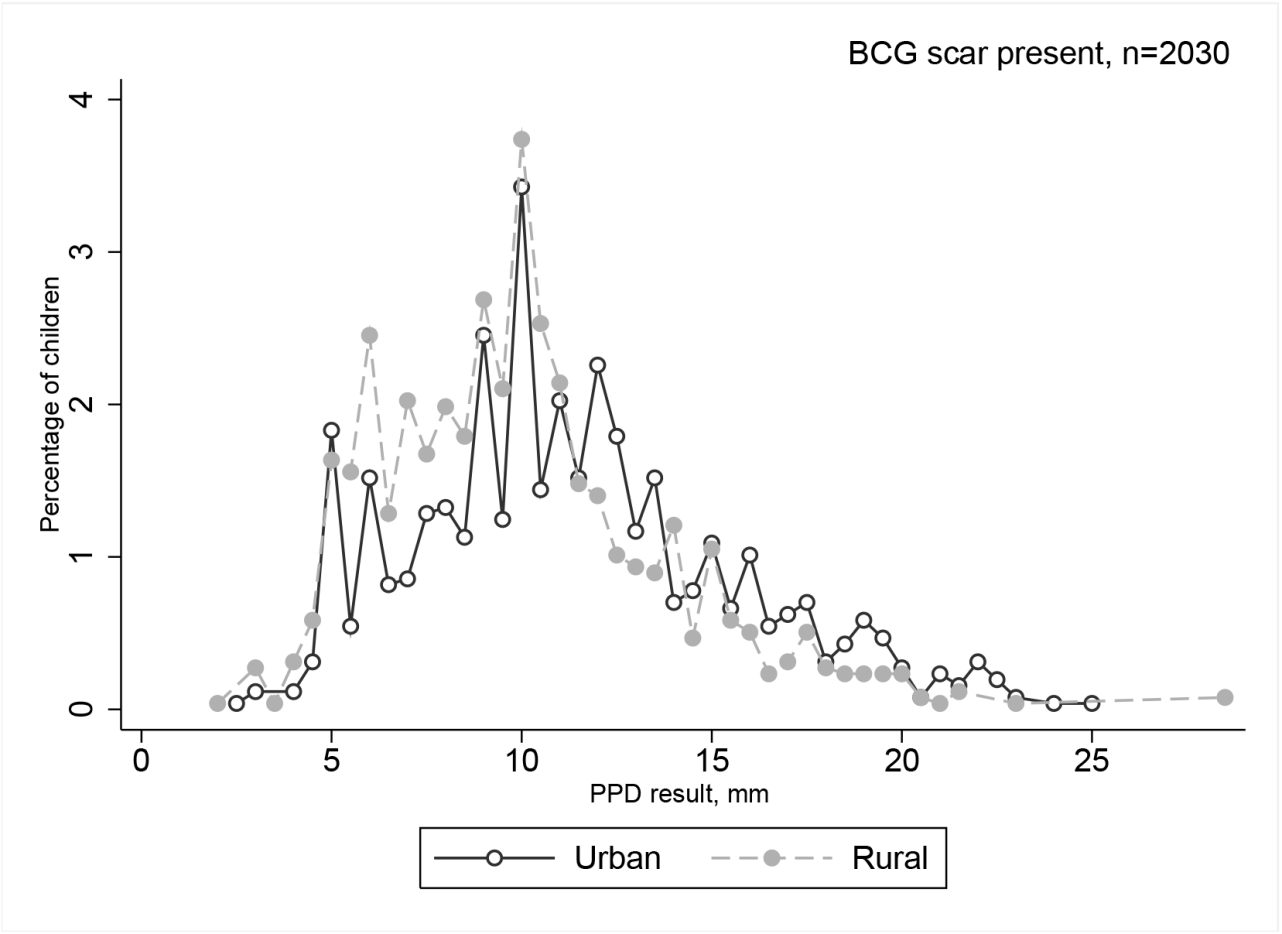
Frequency distribution of tuberculin induration sizes (mm) by residence in school children with a BCG scar present. 10,679 of 13, 386 children, 79.8% had 0 mm induration and are not shown.

**Fig 9:**
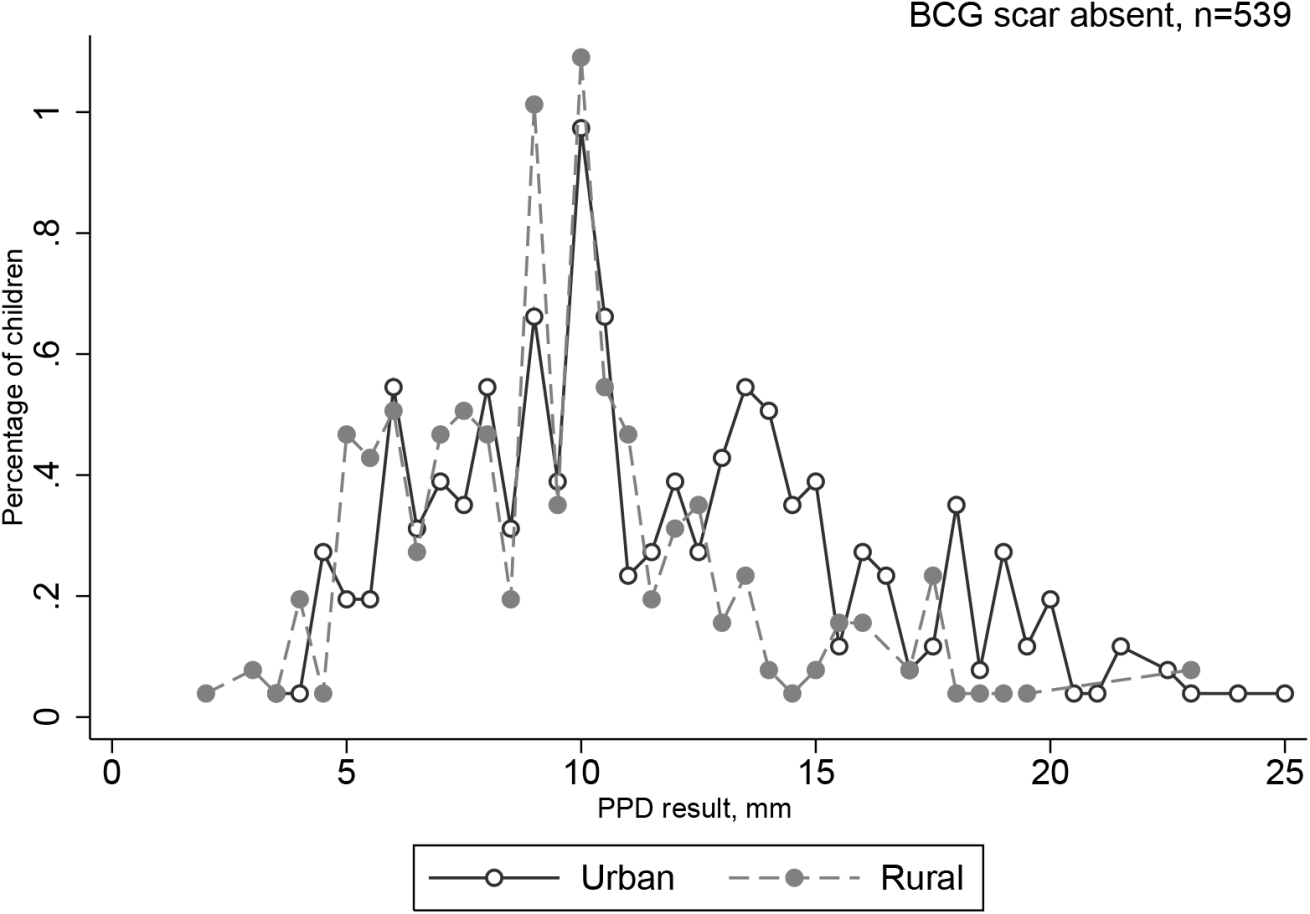
Frequency distribution of tuberculin induration sizes (mm) by residence in school children with a BCG scar absent. Footnote: 10,679 of 13, 386 children, 79.8% had 0 mm induration and are not shown.

Majority, 9,740 (72.8%) of 13,386 children had a BCG scar. **Fig 7** displays the distribution of tuberculin reaction sizes stratified by scar status. Using a 10 mm cut-off for TST positivity, there was a significant difference in the distribution of tuberculin reactions up to an induration of 20 mm (p = 0.02). However, this difference was no longer seen on stratification of TST distributions by residence and BCG scar status (**Figs 8 and 9**) showing urban-rural residence confounds the effect of BCG. There was no difference at the 12 mm (p = 0.21) or 17 mm (p = 0.81). Overall, TST results did not vary significantly by BCG vaccination status (p = 0.14). There was a non-significant trend towards BCG scar as a surrogate for vaccination differing by locality (rural, 23.6 [22.5–24.7]% vs. urban 22.3 [21.3–23.3]%, p = 0.08).

### Estimating the prevalence of LTBI

#### Using TST cut-offs and considering demographic characteristics of participants

The prevalence of LTBI was determined at 10 mm, 12 mm (mixture modelling) and 17 mm (mirror method in TB cases). With the 12 mm cut-off, the prevalence of LTBI was 6.9% (95% CI: 6.5–7.4%), which was lower than the 11.5% (95%CI 11.0–12.1) seen with the 10 mm cut-off. In multivariate analyses, a positive result was associated with age at the 10 mm cut-off, with gender residence at 10 and 12 mm, and urban residence at 12 and 17 mm (see Tables 1–3). Schoolchildren aged 10–11 years were more likely to be TST positive compared to those aged < 9 years at the 10 mm and 12 mm but not at 17 mm (Tables 1–3) cut-offs.

**Table 1:** Prevalence of latent tuberculosis and annual risk of *M*. *tuberculosis* infection in Gambian schoolchildren aged 6–11 years using a 10mm cut-off point.

	Infected	Univariable		Multivariable^b^		ARTI (%)	ARTI (95% CI)
	n/N^a^ (%)	Odds Ratio (95% CI)	p-value	Odds Ratio (95% CI)	p-value		
ALL	1457/12629 (11.5)					1.27	1.09–1.49
Age, years							
6	22/306 (7.2)	1		1			
7	113/1536 (7.4)	1.03 (0.64–1.65)	0.918	1.09 (0.63–1.88)	0.754	1.14	0.96–1.34
8	276/2679 (10.3)	1.48 (0.94–2.33)	0.087	1.34 (0.80–2.24)	0.269	1.01	0.85–1.20
9	310/2825 (11.0)	1.59 (1.02–2.49)	0.043	1.54 (0.92–2.60)	0.099	1.27	1.08–1.49
10	416/2992 (13.9)	2.08 (1.33–3.26)	0.001	2.01 (1.24–3.42)	0.005	1.22	1.04–1.43
11	320/2291 (14.0)	2.10 (1.34–3.29)	0.001	1.98 (1.19–3.31)	0.009	1.42	1.22–1.64
BCG Scar							
Absent	299/2889 (10.4)	1		1		1.13	0.95–1.32
Present	1158/9740 (11.9)	1.17 (1.02–1.34)	0.023	1.16 (0.99–1.35)	0.063	1.31	1.12–1.53
Gender							
Female	721/6651 (10.8)	1		1		1.19	1.01–1.40
Male	736/5978 (12.3)	1.15 (1.04–1.29)	0.010	1.15 (1.01–1.30)	0.035	1.36	1.17–1.58
BMI-for-age							
≥ -2	933/8065 (11.6)	1		1		1.18	1.12–1.25
≤ -2	162/1485 (10.9)	0.94 (0.78–1.12)	0.464	0.92 (0.77–1.10)	0.342	1.28	1.21–1.34
Locality							
Rural	641/5509(11.6)	1		1		1.29	1.09–1.49
Urban	816/7120 (11.5)	0.98 (0.88–1.1)	0.760	0.99 (0.88–1.13)	0.969	1.26	1.07–1.47

95% Confidence Interval (95%CI)

^a^ Denominator, N does not include non-reactive TST

^b^ Adjusted for all other terms in the model

**Table 2:** Prevalence of latent tuberculosis and annual risk of *M*. *tuberculosis* infection in Gambian schoolchildren aged 6–11 years using a 12mm cut-off point.

	Infected	Univariable		Multivariable^b^		ARTI (%)	ARTI (95% CI)
	n/N^a^ (%)	Odds Ratio (95% CI)	p-value	Odds Ratio (95% CI)	p-value		
ALL	873/12629 (6.9)					0.75	0.60–0.91
Age, years							
6	14/306 (4.6)	1		1		0.72	0.58–0.88
7	71/1536 (4.6)	1.01 (0.56–1.81)	0.971	1.13 (0.58–2.20)	0.713	0.63	0.50–0.78
8	162/2679 (6.5)	1.34 (0.76–2.34)	0.302	1.21 (0.65–2.29)	0.541	0.73	0.59–0.89
9	186/2825 (6.6)	1.47 (0.84–2.56)	0.175	1.33 (0.71–2.50)	0.370	0.71	0.57–0.88
10	244/2992 (8.2)	1.85 (1.07–3.21)	0.029	1.77 (0.95–3.30)	0.074	0.81	0.66–0.98
11	196/2291 (8.6)	1.95 (1.12–3.40)	0.018	1.77 (0.94–3.32)	0.076	0.77	0.63–0.94
BCG Scar	** **				** **	** **	
Absent	185/2889 (6.4)	1		1		0.68	0.54–0.84
Present	688/9740 (7.1)	1.11 (0.94–1.31)	0.220	1.13 (0.93–1.37)	0.208	0.76	0.62–0.94
Gender	** **				** **	** **	
Female	422/6651 (6.3)	1		1		0.68	0.54–0.84
Male	451/5978 (7.5)	1.20 (1.05–1.38)	0.008	1.21 (1.04–1.43)	0.013	0.81	0.67–0.99
BMI-for-age							
≥ -2	560/8065 (6.9)	1		1		0.77	0.61–0.97
≤ -2	108/1485 (7.3)	1.05 (0.85–1.30)	0.648	1.04 (0.84–1.29)	0.730	0.75	0.58–0.94
Locality	** **				** **	** **	
Rural	328/5509(6.0)	1		1		0.64	0.51–0.80
Urban	545/7120 (7.7)	1.31 (1.14–1.51)	<0.001	1.34 (1.14–1.58)	0.001	0.83	0.68–1.01

95% Confidence Interval (95%CI)

^a^ Denominator, N does not include non-reactive TST

^b^ Adjusted for all other terms in the model

**Table 3:** Prevalence of latent tuberculosis and annual risk of *M*. *tuberculosis* infection in Gambian schoolchildren aged 6–11 years using a 17mm cut-off point.

	Infected	Univariable		Multivariable^b^		ARTI (%)	ARTI (95% CI)
	n/N^a^(%)	Odds Ratio (95% CI)	p-value	Odds Ratio (95% CI)	p-value		
ALL	234/12629 (1.9)					0.20	0.13–0.29
Age, years					** **	** **	
6	3/306 (1.0)	1		1		0.15	0.09–0.23
7	21/1536 (1.4)	1.40 (0.41–4.72)	0.588	1.31 (0.38–4.50)	0.665	0.18	0.12–0.27
8	41/2679 (1.5)	1.57 (0.48–5.10)	0.453	1.16 (0.35–3.80)	0.810	0.18	0.12–0.27
9	51/2825 (1.8)	1.86 (0.58–5.99)	0.300	1.40 (0.43–4.55)	0.577	0.19	0.12–0.28
10	65/2992 (2.2)	2.24 (0.7–7.18)	0.174	1.71 (0.53–5.52)	0.370	0.21	0.13–0.30
11	53/2291 (2.3)	2.39 (0.74–7.7)	0.144	1.72 (0.53–5.61)	0.368	0.20	0.13–0.30
BCG Scar	** **					** **	
Absent	55/2889 (1.9)	1		1		0.20	0.13–0.29
Present	179/9740 (1.8)	0.96 (0.71–1.31)	0.817	1.00 (0.71–1.40)	0.985	0.19	0.13–0.29
Gender	** **					** **	
Female	114/6651 (1.7)	1		1		0.18	0.12–0.27
Male	120/5978 (2.0)	1.17 (0.91–1.52)	0.223	1.17 (0.88–1.55)	0.285	0.21	0.14–0.31
BMI-for-age							
≥ -2	154/8065 (1.9)	1		1		0.20	0.16–0.22
≤ -2	37/1485 (2.5)	1.31 (0.91–1.89)	0.142	1.30 (0.90–1.87)	0.156	0.26	0.17–0.37
Locality	** **					** **	
Rural	75/5509 (1.4)	1		1		0.14	0.08–0.22
Urban	159/7120 (2.2)	1.65 (1.25–2.18)	<0.001	1.67 (1.22–2.29)	0.001	0.24	0.16–0.34

95% Confidence Interval (95%CI)

^a^ Denominator, N does not include non-reactive TST

^b^ Adjusted for all other terms in the model

#### Estimating of the annual risk of *M*. *tuberculosis* infection (ARTI)

*ARTI estimates using mixture method and fixed TST cut-offs*: The estimated ARTI using all three TST cut-offs are as shown in Tables 1–3. Overall, ARTI was highest using the fixed 10 mm cut-off. With the 12 mm cut-off, which fits our data best, the ARTI estimate is 0.75%. Unsurprisingly, ARTI was least with the mirror method.

#### Nutritional Status and tuberculin reactions

WHOAnthro produced valid WAZ, HAZ and BAZ scores for 6540, 9582 and 9550 participants. The prevalence of underweight children was 11.2% (732 of 6540) and for severe underweight, 2.4% (158 of 6540). There were significantly more underweight children in rural compared to urban areas (13.6% [95%CI: 12.2–14.9) vs. (9.6% [8.7–10.6] respectively, p <0.0001). The same pattern was seen for severe underweight (3.7% [95%CI: 3.0–4.4] vs. 1.6% [1.1–2.0] respectively, p <0.0001).

The prevalence of stunting was 7.4% (710 of 9582) and 1.3% (127 of 9582) for severe stunting. There were twice as more stunted children in rural (10.3% [95%CI: 9.3–11.2]) compared to urban areas (5.4% [95%CI: 4.8–6.0], p <0.0001). Severely stunted children were more than 2.7 times more likely to be inhabitants of rural (2.1% [95%CI: 1.6–2.5]) compared to urban areas (0.79% [95%CI: 0.6–1.0], p<0.0001)

BMI-for-age did not differ by urban or rural residence and the prevalence of severe thinness, thinness, overweight and obesity were 2.7% (260), 12.8% (1225), 1.8% (171) and 0.4% (41 of 9550) respectively.

In univariable analyses, WAZ, HAZ and BAZ were not associated with the categories of TST induration sizes and there were no significant differences in the mean sizes of indurations when only indurations >0 mm were considered. In multivariable logistic regression analyses, underweight children were less likely to be positive for LTBI at the 10 mm cut-off (Odds Ratio, OR 0.68 [95%CI 0.49–0.95], p = 0.023) but this effect was not seen with HAZ, BAZ and at the 12 and 17 mm cut-offs. BCG scar status and residence did not have an effect. Using 12 mm and 17 mm definitions for LTBI and adjusting for WAZ, HAZ, BAZ and BCG scar status, urban residents were more likely to be TST positive (OR 1.35 [95%CI: 1.1–1.67], p = 0.006 and OR 1.72 [95%CI: p = 0.010 respectively). There were no interactions between WAZ, HAZ and BAZ.

Tables 1–3 show overall adjusted estimates of ARTI adjusted for BAZ, age, gender, BCG scar status and residence.

## Discussion

Although there have been some earlier studies in The Gambia, the results presented here are from the first nationwide tuberculin survey of schoolchildren following recommendations in current guidelines. [1] Roelsgaard, et al, [5] found LTBI prevalence of 12% and 11% in urban and rural Gambia respectively for children aged 0–9 years at a 10 mm cut-off. Although similar to our results, 11.5% at a 10 mm cut- off, it is not directly comparable because 1 TU PPD was used. TST induration sizes are affected by PPD strength.[25, 26] In 1976, others found a 19.7% LTBI prevalence at 10 mm using the same 2 TU/ PPD RT-23 [6] as us. However, the population surveyed were much older children with more cumulative TB exposure. About 15 years later, a survey that included 6–11 year olds, found 42% LTBI at the same 10 mm cut-off and only 34.7% had BCG scars.[8] The ARTIs from these earlier surveys were 3.05, 1.70, and 6.21 in 1958, 1976 and 1994 respectively. It would appear that LTBI and ARTI levels have now declined at least compared to those seen in the 1994 at the 10 mm TST cut-off. Unfortunately, any interpretation of trends is limited by differences in methodology of TST surveys and the absence of concurrent TB disease prevalence data.

Addo, et al in Ghana found prevalence of LTBI at 17mm was <2% and ranged from 8.2–11.8% for the 10 mm cut-off. A 15 mm cut-off, as expected, gave a lower prevalence of LTBI (0.0–5.4%) and ARTI (0.0–0.6%) than for our 12 mm cut-off. Historical data from Benin Republic gave lower LTBI and ARTI estimates (3.7% and 0.47%) with a lower burden of active TB disease. However, authors used alternative methods for derivation of cut-offs for definitions of LTBI.[27] A recent survey in the Central Africa region reported similar ARTIs using 10 mm (1.9%) and 15 mm (0.8%) cut-offs.[28]

In this study, LTBI prevalence was associated with urban residence. This is consistent with the 2012 TB prevalence survey that reported a higher prevalence of bacteriologically confirmed TB in urban compared to rural residents (266 vs.169 per 100,000 population ≥15 years, [Adetifa I. Submitted]). In addition, males had significantly more LTBI. In addition to an increased likelihood of developing hypersensitivity to mycobacterial antigens, post pubertal males are also thought to have more LTBI than females because of increased TB exposure. [29]

At the 10 mm cut-off, ARTI was identical (1.1–1.5%) for urban and rural areas and lower (0.5–1.0%) for rural and urban areas for the mixture method estimate. Surveys in West Africa and elsewhere have found the 10mm cut-off over-estimates LTBI and ARTI. [14, 18] This suggests the 10mm threshold has less specificity for detection of LTBI. It is also possible a larger proportion of children in rural areas have intermediate TST reactions due to a higher exposure to Non-Tuberculous Mycobacteria (NTM) exposure compared to those in urban areas. Perhaps this contributes in part to the difference in urban-rural comparisons depending on the TST cut-off point. The 12 mm cut-off derived from mixture modelling appears to be the most useful for determining LTBI prevalence and ARTI.

With our data, we were unable to identify a single and valid TST cut-off as no clear bimodal distribution was seen. Considering this and the difference between distributions of TST sizes at the 10 mm cut-off, we are unable to eliminate the contributions of non-specific reactions resulting from exposure to non-tuberculosis mycobacteria in the community and BCG vaccination.

ARTI is the average of annual risks of infection experienced from the birth to the survey. Since the average age of the children was 9 years, our estimated ARTI most likely corresponds more closely to the 2006–7 period. Although other surveys report age dependent ARTI rates [14, 30], ARTI estimates here do not vary significantly with age. If ARTI declined over time and increases with age, the apparent effect in a single survey may be independent of age. Unfortunately, we are unable to dissect age effects from calendar year effects with a single survey.

Similar to others, we found BCG scars in only 73% of school children. [7, 31] Given the high vaccination coverage rate, the risk of misclassification of BCG vaccination status is very low. In addition, waning BCG scars and absence of scarring in a variable proportion of BCG vaccinated children are both well-described phenomena. [32] Other surveys have found BCG vaccination has little or no effect on TST results in children. [14, 27] We saw a slightly higher prevalence of LTBI with the presence of a BCG scar but the absolute differences were very small (1.5% at 10 mm and 0.7% at 12 mm). The similarity between the frequency distributions of tuberculin sizes by BCG scar status supports our use of mixture modelling to estimate LTBI prevalence.[33] Tuberculin reaction sizes above 10 mm cut-off are thought to be more valuable for this purpose. [34]

Both anergy and/or lower TST induration sizes resulting from the immunosuppressive effect of severe malnutrition can cause false negative TST results.[35] Not many TST-surveys have examined the impact of nutritional status on survey results. It was not surprising to see the higher burden of malnutrition in rural areas across all the anthropometric indices. Contrary to expectations, there were no differences in the proportions of non-reactive TSTs or mean sizes of tuberculin indurations by nutritional status. Underweight children were less likely to be TST positive at the 10 mm cut-off suggesting the possibility of underestimating LTBI if 10 mm was found to be the best fit for our data. However, we found no effect for malnutrition at the 12 and 17 mm cut-offs.

Un-enrolled children are likely to have a higher risk of TB because they tend to come from lower socio-economic classes. Because primary school enrolment increased from 52% to 61% and to 90% in 2000, 2006 and 2011 respectively (Ministry of Basic and Secondary Education, Republic of The Gambia), we do not think we missed many of these children. However, as we did not collect socioeconomic data, we did not test for associations with LTBI and ARTI.

While this survey provides an important baseline to estimate trends in future surveys, the largely overlapping distributions seen with mixture modelling and difficulty in determining LTBI prevalence suggests future TST surveys may not be very helpful. Should they been deemed necessary, interferon gamma release assays (IGRAs) may provide complementary results to increase the utility of survey outcomes. Although our host community’s attitudes and beliefs around blood and sampling for research, the requirement for venepuncture, even for a subset of otherwise healthy school children would substantially reduce participation in such a survey. Furthermore, there are issues of cost and the logistical challenge of getting blood samples to the laboratory for analyses especially whilst working in distant locations. Finally, our experience with IGRAs in The Gambia suggests they do not have any sensitivity or specificity benefit over the TST. [36, 37] These arguments will become moot if newer but more sensitive and specific diagnostic tools for LTBI become available.

## Conclusion

Our survey showed a relatively low prevalence of LTBI and estimated ARTI. LTBI was more prevalent in urban compared to rural areas and overall, malnutrition had no impact on LTBI estimates for the 12 mm cut-off, which was the best fit for our data.
